# Teriparatide in the Treatment of Severe Postmenopausal Osteoporosis: A Cost-Utility Analysis

**DOI:** 10.22037/ijpr.2019.1100679

**Published:** 2019

**Authors:** Saeed Taheri, Fatemeh Mirzayeh Fashami, Farzad Peiravian, Nazila Yousefi

**Affiliations:** *Department of Pharmacoeconomics and Pharma Management, School of Pharmacy, Shahid Beheshti University of Medical Sciences.*

**Keywords:** Teriparatide, Cost-effectiveness, Osteoporosis, Economic evaluation, Cost-utility

## Abstract

Teriparatide is a new agent serves as a treatment of choice for severe post-menopausal osteoporotic patients who are at high risk of fracture or have failed or been intolerant of previous osteoporosis therapy. The objective of this study is to estimate the cost-utility of teriparatide compared with no treatment from health system perspective in Iran. A micro-simulation model was developed for a cohort of hypothetical Iranian patient population (women aged 70 years, T-score -2.5 with previous fracture or T-score -3.0 without prior fracture) over a lifetime horizon. The model consisted of the seven health states. During each cycle, patients could have a fracture, remain healthy, remain in a post-fracture state or die. Background fracture risks, mortality rates, persistence rates, utilities, medical and drug costs were derived using published sources. Total accumulated life-time costs and quality-adjusted life years (QALYs) were estimated. Teriparatide was associated with 4.786 QALYs and total direct costs of IRR 143,168,259 over a lifetime horizon. Compared to no treatment, teriparatide provided an additional 0.145 QALY at an incremental cost of IRR 33,511,013. The resulting incremental cost-effectiveness ratio was IRR 230,333,030/QALYs gained. The probabilistic analysis showed that accepting a willingness-to-pay 2 and 3 GDP/capita in Iran, the probability of teriparatide being cost-effective were 51% and 83%, respectively. Compared to no treatment, teriparatide was indicated to be more costly and associated with fewer fractures, more life-years, and more QALYs. The result showed that teriparatide may be considered a cost-effective intervention when targeted to the appropriate patients.

## Introduction

Considering the fact that today’s population is aging as an inevitable consequence of a steady increase in life expectancy (1), osteoporosis has now become a global widespread disease (1). Although osteoporosis affects both sexes, the prevalence of the disease is significantly higher in women of all races, especially in their postmenopausal stage (2, 3). It is estimated that about 200 million women worldwide suffer from the disease (4). In the USA and Europe, osteoporosis affects around 30% of post-menopausal women of whom 40% experience one or more bone fractures in their life (5). 

In Iran, as an upper-middle income country, the incidence of osteoporosis is growing. The population is graying with more than 4 million people over the age of 65 (6, 7). In addition, 45% of men and 61% of women have been reported to suffer from vitamin D deficiency, which is a well-known risk factor for osteoporosis (8, 9). Among people older than 50 years, the incidence of osteoporosis is over 22% for women and about 11% for men. Considering a large number of patients with osteoporosis in Iran, around two million people have been reported to be at the risk of fracture (10, 11). 

Teriparatide is the only anabolic treatment available for osteoporosis. Unlike all the antiresorptive medicines which reduce bone resorption, teriparatide has a different mechanism and triggers bone formation (12). According to the guidelines of American Association of Clinical Endocrinologists and American College of Endocrinology (AACE/AAE), teriparatide is a treatment of choice in postmenopausal osteoporosis (PMO) patients who are at high risk of fracture or have failed or have not responded to previous osteoporosis therapies (13, 14). 

There are a few local cost-effectiveness studies available to compare different treatments in the management of osteoporosis; however, there is not any local study evaluating the cost and benefits of teriparatide in the target group of patients, i.e. patients with severe osteoporosis only. This study was aimed to assess the cost-utility of teriparatide in comparison with no treatment in severe PMO from the health system perspective in Iran.

*Methods*

*Model overview*

**Figure 1:**
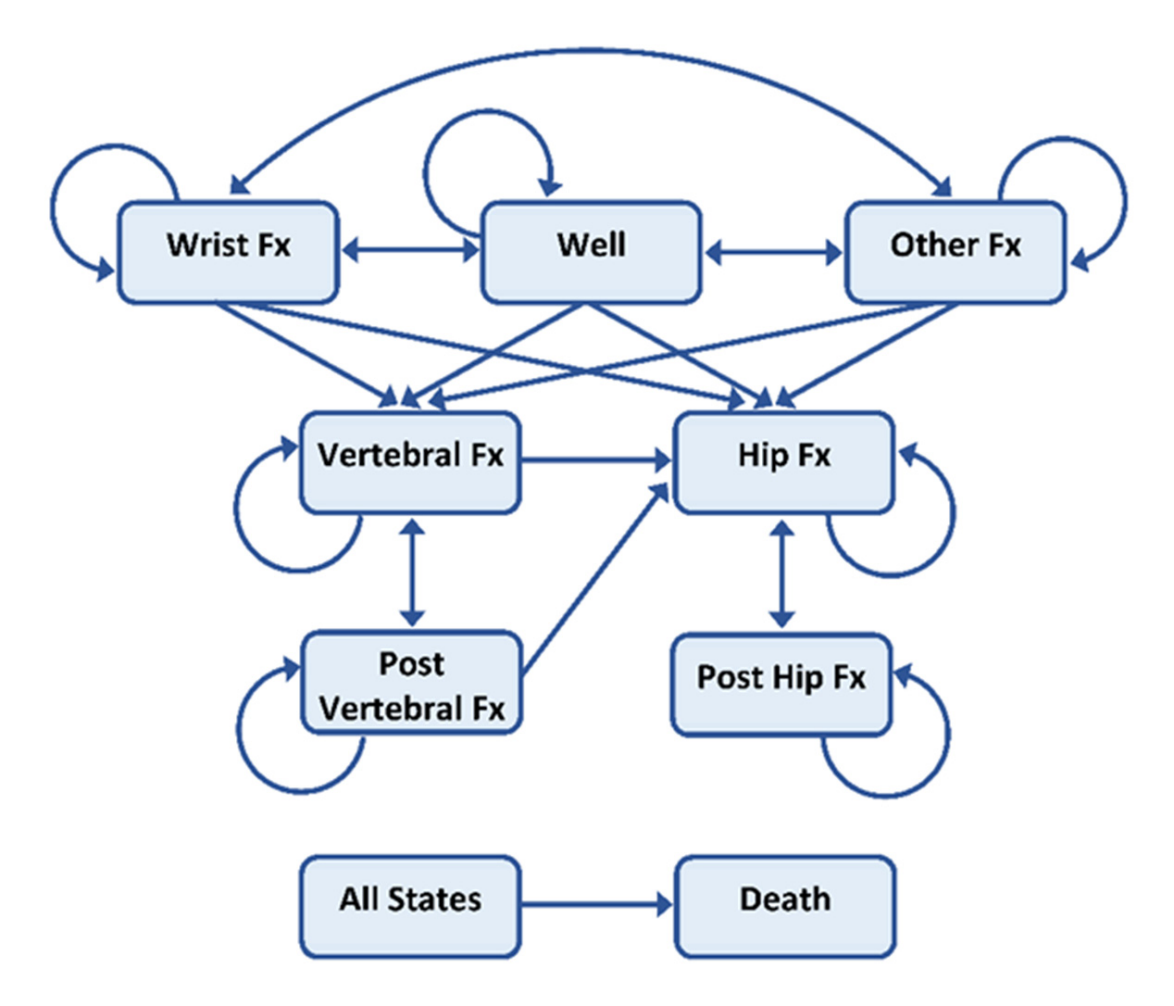
Health state structure of Markov model

A seven health state Markov cohort micro-simulation model was developed to investigate the cost-effectiveness of teriparatide in the treatment of PMO compared to placebo using TreeAge Pro 2018 Software. The model was developed based on the previously published lifetime Markov cohort models in PMO (15–17) to encompass the results of teriparatide outcome and cost data for 2018. Figure 1 shows the structure of the Markov model. 

The cycle length was 6 months, and all patients were followed through the model from their age at the initiation of the treatment until they were 100 years old or dead. All patients began the simulation in the no-fracture health state. For each six-month cycle, i.e. Markov cycle, patients in the cohort were assigned a probability of staying healthy, sustaining a fracture or dying. Patients in the cohort who experienced a fracture may transit to a hip fracture, vertebral fracture or other osteoporotic fracture depending on the fracture type. After one year in a given fracture state, the patients can experience 4 states: sustain a new fracture, move to the post-fracture state (either post-hip or post-vertebral fracture, depending on the previous health state), move back to the healthy state (for “other fracture group” only) or die. Patients in the post-vertebral fracture state can stay in this state, experience a new vertebral fracture, experience a new hip fracture or die. From the post-hip fracture state, patients can remain in this state, sustain a new hip fracture or die. The patient cohort was adjusted according to their baseline characteristics, i.e. if the patients had a prevalent fracture before the intervention, their quality of life, costs involved, as well as their mortality risk would be adjusted accordingly.

*Clinical data and treatment effect*

The model captured the effectiveness of teriparatide in severe PMO patients, the ones with the mean age of 70 years with either a T-score of -2.5 and a prior fracture as the patient population. In keeping with other similar studies, the treatment duration with 20µg daily teriparatide was assumed to be 18 months (15, 18). 

There are sufficient clinical data available about teriparatide use in PMO such that clinical effectiveness, the relative risk of fracture, the incidence of osteoporotic fracture, as well as the risk of relevant adverse effects are extracted from the relative studies (12).

According to Neer *et al*., treatment with teriparatide reduced the risk of hip fracture (RR 0.50, CI 0.09–2.73), wrist fracture (RR 0.54, CI 0.22–1.35), humerus fracture (RR 0.80, CI 0.22–2.98), clinical vertebral fracture (RR 0.35, CI 0.22– 0.55), and other non-vertebral fracture (RR 0.47, CI 0.25–0.88)(12, 17).

In line with treatment guidelines, patients were assumed to be given teriparatide for 18 months, and the effectiveness was assumed to apply at its maximum initial level for the whole treatment period (15). Thereafter, the effect was assumed to taper to zero over 24 and 30 months (i.e., the offset time of treatment effect) for vertebral and non-vertebral fractures respectively, based on the follow-up study of the clinical trial (12, 19). This approach had been formerly adopted in similar cost-effectiveness studies (15, 18). In a sensitivity analysis, treatment periods of 12 and 24 months and scenarios assuming no and doubled offset time were explored.

*Risk of fracture*

As only age-specific hip fracture incidence rate was available for Iran (20–23), other, age-specific fracture risks were derived from various studies (16, 23–25). Age-specific wrist fracture, vertebral fracture, and non-vertebral fracture risks were only available for a Swedish population (25). Therefore, to assess vertebral fracture risks in Iran, it was presumed that the ratio of clinical vertebral fracture to hip fracture was similar to the ratio in Sweden. Thusly, the vertebral fracture risk in Iran was calculated based on this ratio and hip fracture risk in Iran (24). The incidence of fractures is illustrated in Table 1. The incidence data were linearly extrapolated to 100 years of age where data were missing.

The risk of having an osteoporotic fracture prior to a fracture at the same location was considered based on a study by Johnell in 2004. Their study showed that the risk of upcoming fracture increases for 5 years after a fracture and it is at the highest level in the first year (26).

*Mortality*

The age-specific baseline mortality in Iranian women was derived from WHO data(27) and was subsequently applied to the relative risk of mortality after a fracture, which is illustrated in table 2 (28).

As it was revealed in the previous studies, mortality in the first year following a fracture was assumed to be higher than in subsequent years for hip and vertebral fractures. Other osteoporotic fractures were assumed to only have an increased risk of mortality in the first year of fracture (26, 29, 30). 

*Persistence rate*

Although teriparatide needs to be injected twice daily and persistence rate is not anticipated to be high, Landfeldt and his colleagues reported that persistence rate to the medicine is 70.3% (Cl 95 64.0-75.8%) for 1 year (31). The same persistence rate was used in this study. 

*Quality of life and utility*

The background age-specific utility weights were derived from the available literature (17, 32, 33). Based on the published clinical studies, it was assumed that each fracture decreases the quality of life (QoL) at a certain level (34, 35). The impact of hip and vertebral fracture on quality of life was extracted from a meta-analysis (3). The disutility associated with “other” fractures in the first year was derived from a study by Borgstrom *et al*. (4). The fracture-specific utility multipliers are shown in Table 3.

For hip and vertebral fractures, reduction in QoL was taken into account both in the short term and the long term based on the fact that utility decrease is different in the first year compared to the following years; however, in wrist fractures, the utility decrease was considered for the first year only. The QoLs were calculated by multiplying the certain age-specific QoL by the relating fracture-specific utility multipliers.

*Costs and discounting*

According to the study perspective, health system perspective, all direct medical costs were taken into account in the year 2018 values at the average governmental exchange rate of 1 US dollar = 42,000 Iranian Rial (IRR). Direct costs included costs of medicines, healthcare, and hospitalization. Patients were considered to be hospitalized in the ICU department for 3 to 5 days for each fracture. Also, the nursing costs, physicians’ visit, and laboratory tests were included. Costs of medicines were extracted from the official prices published by the Food and Drug Administration of Iran (36).

Cost items of hospitalization for hip, vertebral, wrist, and other fractures were identified based on the routine practice of key opinion leaders (KOLs) in osteoporosis treatment in Iran as well as the available literature. The total cost of specific fracture management was extracted from Iranian osteoporosis society and Iranian rheumatology association including the cost of surgery, the cost of hospitalization, the cost of monitoring, the cost of physicians visit, and the cost of post-surgery follow-up. Then cost calculation was done based on the official prices for healthcare in Iran (37). All the cost related data are shown in table 4 and 5 (38, 39). 

In the long term basis, costs of 6 months of nursing at home and annual bone marrow density (BMD) test were entered into the model. For hip and vertebral fracture, the cost of managing bed sores was also taken into account. Both groups were considered to take at least 1000 mg Calcium Carbonate and 400 IU vitamin D once daily along with tablet of Naproxen 500 mg twice daily. All the costs are shown in table 4 and 5. Costs and effects were discounted at a rate of 7.2% and 5% respectively (40).

Cost of teriparatide in this study is calculated based on locally produced biosimilar product (CinnoPar®, CinnaGen Company).

*Sensitivity analysis*

One-way deterministic sensitivity analyses were carried out to investigate the impact of individual variables on the results of the model. Variables were changed over a credible range of probabilities, parameters, and assumptions extracted from the literature or an assumed variation of ± 25% around the base case. Sensitivity results were plotted on a Tornado diagram by ranking parameters from the most sensitive to the least sensitive. 

Furthermore, a probabilistic sensitivity analysis (PSA), based on a second-order Monte-Carlo simulation, was carried out to include uncertainties of all parameters concurrently. The number of iterations was equal to 1000. The results of Monte-Carlo simulation were presented in the form of incremental cost-effectiveness scatterplot, which illustrates the proportion of samples that are below different values of willingness to pay (WTP) for a QALY gained. In this study, the acceptable WTP threshold was considered up to the triplicate of Gross Domestic Production (GDP) per capita according to the World Health Organization (WHO) recommendation(41). GDP/Capita in Iran was considered IRR 138,000,000 (USD 3,285) for the 2017 fiscal year. As a consequence, a cost-effectiveness acceptability curve was determined over a range of WTP thresholds, from IRR 138,000,000 to IRR 414,000,000 per QALY. Fracture relative risks, utilities, and health-care costs were assumed to have log-normal, beta, and gamma distributions respectively considering their standard deviation (Table 6).

*Outcomes*

Quality-adjusted life years (QALYs), the gold standard outcome measurement for cost-utility studies, were used to measure the health benefits delivered by teriparatide treatment regimen. QALYs were calculated by downwardly adjusting the life expectancy of the treatment for losses in quality of life as measured by utility. Incremental cost-effectiveness ratio (ICER) was calculated by dividing the difference in costs (𝞓C) by the difference in effectiveness between two competing strategies (𝞓E).

 ICER=$\frac{\mathrm{\Delta C}}{\mathrm{\Delta E}}$=$\frac{C1-C0}{E1-E0}$                    Equ.1 

## Results

*Base-case results*

Total discounted costs and QALYs were estimated to be IRR 139,861,307 and 4.67 for teriparatide versus IRR 104,586,853 and 4.53 for no treatment respectively from the health system perspective, utilizing first order Monte-Carlo simulation (microsimulation). Compared to no treatment, the costs associated with teriparatide were IRR 35,274,454 higher but teriparatide had a 0.138 additional QALY per patient over a lifetime horizon. The ICER was calculated to be IRR 254,750,619. If we go by the WHO recommended threshold for countries, teriparatide could be considered as a cost-effective treatment in severe PMO in comparison to no treatment.

**Figure 2:**
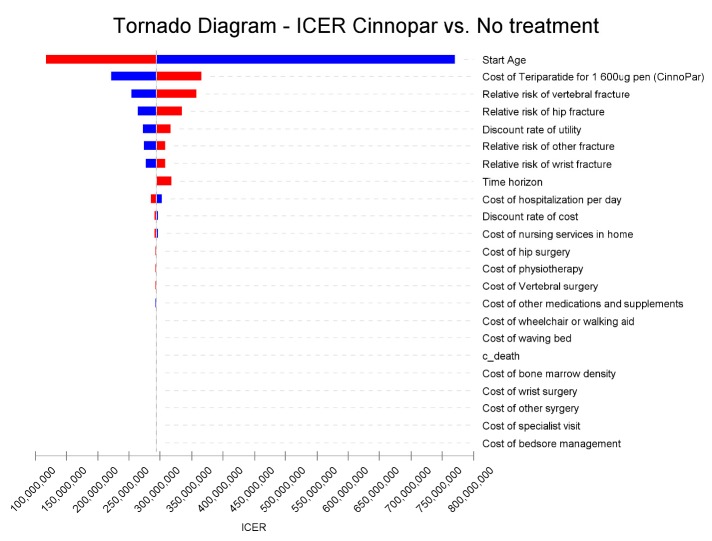
One-way sensitivity analysis tornado diagram: teriparatide vs. no treatment analyses of changes in the key variables around± 25%.

**Figure 3:**
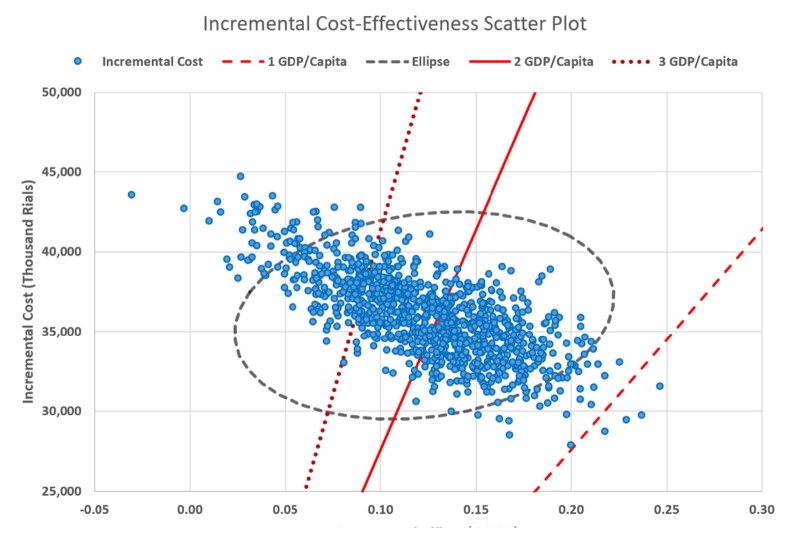
Results of Monte-Carlo simulation with a willingness-to-pay threshold of 1 to 3 GDP/Capita

**Figure 4:**
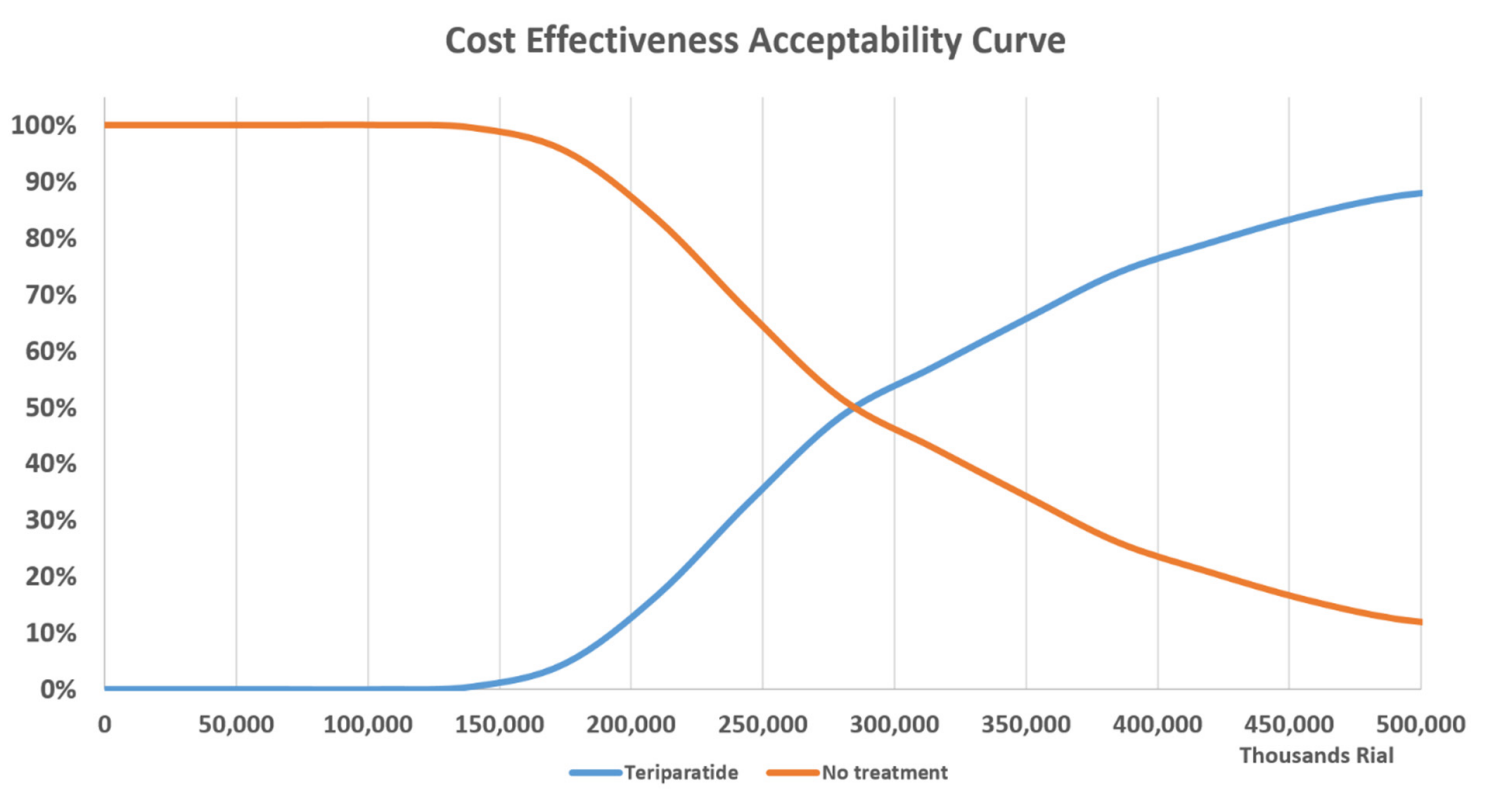
The cost-effectiveness acceptability curve for teriparatide vs. no treatment

**Table 1:** Incidence of osteoporotic fractures (per 1000 women) in Iran

**Age group**	**Hip**	**Wrist** *	**Vertebral** *	**Other non-Vertebral** *
**50–54**	0.39	4.18	1.61	1.39
**55–59**	0.65	4.56	1.58	2.25
**60–64**	1.57	5.68	2.73	1.67
**65–69**	2.53	6.91	3.98	2.47
**70–74**	4.70	9.04	7.39	5.08
**75–79**	9.07	10.33	11.11	8.39
**80–84**	15.02	12.08	11.07	15.18
**85+**	24.60	13.23	14.1	27.84

* Estimated based on the ratio of fractures in Sweden.

**Table 2:** Relative risk of mortality after different fractures in women

**Age**	**Hip fracture (1st year)**	**Clinical vertebral fracture (1st year)**	**Hip fracture (Subsequent years)**	**Clinical vertebral fracture (Subsequent years)**	**Other**
70-74	5.91	6.47	4.55	4.23	4.06
75-79	4.46	4.63	3.42	3.01	2.94
80-84	3.37	3.31	2.57	2.14	2.12
85-89	2.55	2.37	1.94	1.53	1.53
90-94	1.93	1.69	1.46	1.09	1.11

**Table 3:** Multipliers of utility by fracture type and adverse events

**Fracture type/period**	**Utility multiplier**	**Source**
First year after fracture		
Hip fracture	0.792	(34)
Clinical vertebral fracture	0.626	(34)
Wrist fracture	0.977	(34)
Second year and following years after fracture		
Hip fracture	0.9	(34, 35)
Clinical vertebral fracture	0.93	(34, 35)

**Table 4:** Medical costs of fracture management

**Type of fracture**	**Number of Physician visit in 3 months**	**Mean Hospitalization Duration**	**Surgery (yes/no? and proportion among patients)**	**Total mean costs in governmental public service (Rs)**	**Total mean costs in private service (Rs)**
**hip fracture**	4	21 days	yes 85%	150,000,000	400,000,000
**vertebral fracture**	5	18 days	yes 60%	100,000,000	250,000,000
**wrist fracture**	2	7 days	yes70%	80,000,000	180,000,000
**other fracture**	2	7 days	yes 90%	80,000,000	180,000,000

**Table 5:** Other medical costs

**Cost Item**	**Cost in IRR**
**Cost of bed sore management during 6 months**	4,000,000
**Cost of bone mineral density test**	1,400,000
**Cost of Cinnopar® 600 mcg in 1 Pen**	4,400,000
**Cost of bed/day hospitalization in ICU**	8,000,000
**Cost of bed/day hospitalization in ward**	4,000,000
**Wheelchair or walking aid**	10,000,000
**Cost of Calcium + vitamin D and naproxen for 6 month**	1,300,000
**Cost of monthly nursing in home during 6 month**	60,000,000
**Cost of physiotherapy**	10,500,000
**Cost of specialist visit**	450,000
**One-off cost for waving bed**	10,000,000

**Table 6:** Distribution of variables for probabilistic sensitivity analysis

**Variable**	**Distribution**	**Lower value**	**Upper value**
Start Age	Normal	60	80
Cost of Hip Surgery	Gamma	28000000	55000000
Cost of Other Surgery	Gamma	14000000	26000000
Cost of Vertebral Surgery	Gamma	37000000	67000000
Cost of Wrist Surgery	Gamma	14000000	26000000
Relative Risk of Hip Fracture with teriparatide	log-normal	0.09	2.73
Relative Risk of Other Fracture with teriparatide	log-normal	0.25	0.88
Relative Risk of Vertebral Fracture with teriparatide	log-normal	0.22	0.55
Relative Risk of Wrist Fracture with teriparatide	log-normal	0.22	1.35
Utility multiplier for Hip fracture (1st year)	Beta	0.66	0.72
Utility multiplier for Hip fracture (2nd year)	Beta	0.76	0.83
Utility multiplier for other fracture	Beta	0.84	0.9
Utility multiplier for Vertebral fracture (1st year)	Beta	0.53	0.64
Utility multiplier for Vertebral fracture (2nd year)	Beta	0.88	0.97
Utility multiplier for wrist fracture	Beta	0.87	0.92

**Table 7:** Base-case results and one way sensitivity analyses of key parameters

**Scenario**	**Total cost (IRR)**	**Incr. cost (IRR)**	**Total effect (QALYs)**	**Incr. effect (QALYs)**	**Incr. cost per QALY gained**
**Base case**					
**Teriparatide No treatment**	139,861,307 104,586,853	35,274,454	4.667 4.528	0.138	254,750,619*
**Sensitivity analyses**					
**Cost of teriparatide decreased 25%**					
**Teriparatide No treatment**	136,036,650 110,782,967	25,253,682	4.667 4.528	0.138	181,681,173*
**Cost of teriparatide increased 25%**					
**Teriparatide No treatment**	150,654,978 108,312,325	42,342,653	4.667 4.528	0.138	304,623,403¥
**Reduced time horizon (5 years)**					
**Teriparatide No treatment**	91,610,564 54,333,485	37,277,080	2.780 2.734	0.045	823,309,766¶
**Reduced time horizon (10 years)**					
**Teriparatide No treatment**	117,982,965 81,786,245	36,196,720	4.025 3.942	0.083	437,711,114¥
**RR of Fracture by teriparatide increased 25%**					
**Teriparatide No treatment**	144,936,562 106,406,933	38,529,628	4.621 4.519	0.102	378,962,544¥
**RR of Fracture by teriparatide decreased 25%**					
**Teriparatide No treatment**	141,229,718 105,425,696	35,804,022	4.782 4.621	0.161	222,435,593*
**Mean age of 60 years for baseline population**					
**Teriparatide No treatment**	123,376,172 81,514,501	41,861,671	6.384 6.335	0.049	854,973,501¶
**Mean age of 80 years for baseline population**					
**Teriparatide No treatment**	169,295,276 141,330,023	27,965,253	3.688 3.527	0.161	174,236,949*
**No discounting**					
**Teriparatide No treatment**	208,932,440 169,149,850	39,782,590	6.159 5.962	0.197	201,445,252*

* Cost-effective at a willingness-to-pay threshold of 2 GPD/capita (i.e. IRR 276,000,000)

¥ Cost-effective at a willingness-to-pay threshold of 3 GPD/capita (i.e. IRR 444,000,000)

¶ Not cost-effective.

*Sensitivity analyses*

Figure 2 *is the tornado diagram, plotted using the results of one-way sensitivity*

The result of the tornado diagram demonstrates that the most important variables in the study are the mean start age of patients and the cost of teriparatide. Results for the sensitivity analyses are demonstrated in Table 7.

The second order Monte-Carlo simulation (PSA) together with micro-simulation resulted in a mean cost for teriparatide of IRR 142,192,356 (95% CI: ± IRR 3,608,728) and for no treatment of IRR 106,043,442 (95% CI: ± IRR 3,268,423). Effectiveness was also robust, with the PSA plus micro-simulation resulting in 4.706 QALYs for teriparatide (95% CI: ± 0.096) and 4.583 for no treatment (95% CI: ± 0.092). The PSA found that in 51% and 81% of iterations teriparatide was cost-effective compared to no treatment at the WTP threshold of 2 and 3 GDP/capita respectively. The results of Monte-Carlo simulation is shown in Figure. 3 as an incremental cost-effectiveness scatter plot.

The cost-effectiveness acceptability curve in Figure 4 illustrates a comparison of treatment with teriparatide vs. no treatment and variation of the WTP threshold from 0 to 3 GDP/capita in Iran per QALY gained, showing that teriparatide is more likely to be cost-effective as the willingness-to-pay threshold increases. As shown in the diagram, at a willingness-to-pay of 2 GDP/capita, the probability of teriparatide being cost-effective would be more than 51%.

## Discussion

To the best of our knowledge, this is one of the first cost-effectiveness studies undertaken to assess teriparatide in the treatment of severe PMO utilizing a micro-simulation Markov cohort model in developing countries. The model was a refinement of previously developed osteoporosis models (16–18, 25, 30, 42–44), updated in light of recent epidemiological evidence indicating that fractures have an important impact on mortality and risk of new fractures, particularly immediately following the fracture (18). In light of the fact that a healthcare budget is tight, a decision analysis like this study may provide healthcare decision makers with the requisite insights to make informed choices. 

The results of the model indicated that, although the total medical costs of teriparatide treatment were higher compared to no treatment over a lifetime horizon, patients receiving teriparatide benefited from more QALYs. Thus, from the health system perspective, the analysis showed that in severe PMO patients, teriparatide would be a cost-effective treatment. 

As has been noted, the model was most sensitive to acquisition costs for teriparatide as well as the mean start age of cohort. Changes in other parameters had no major impact on the results. Minimal effects on the results were observed for other key parameters such as other medical costs, the relative risk of fracture, and discount rates. 

The PSA indicated that the model is robust based on the uncertainty and distribution of parameters such as clinical efficacy relative risk, costs, and utilities. In addition, taking health system perspective into account, the PSA demonstrated that teriparatide was cost-effective in 51% of cases accepting a WTP threshold of 2 GDP/capita and cost-effective in more than 81% of cases accepting a WTP threshold of 3 GDP/capita. Considering the fact that AACE/AAE guideline recommends teriparatide in PMO patients with severe osteoporosis who are at risk of fracture and have failed or been intolerant to the previous treatments, cost-utility of teriparatide were studied in severe PMO patients only.

Cost-effectiveness of teriparatide had been assessed in limited studies. In some studies, teriparatide was not evaluated only in severe osteoporotic patients, for instance, in a study by Parthan *et al*. cost-effectiveness of denosumab was compared to the other medications such as teriparatide. However, all study participants were men above 75 and the inclusion criteria were not defined to include severe post-menopausal osteoporotic patients (30). 

 Although there is an economic evaluation comparing teriparatide versus alendronate in Iran in which authors concluded that teriparatide is not a cost-effective medicine, the result is doubtful due to several methodological issues in that study (45). In the mentioned study, teriparatide was compared with alendronate which was not an appropriate comparator, because according to the AACE/AAE guideline, teriparatide is the treatment of choice in PMO patients who have failed or been intolerant of previous osteoporosis therapy-including Alendronate (13, 14). Over and above, Azar *et al*. compared the cost-effectiveness of teriparatide in women aged 60 without any evaluation of the fracture risk and limitations relating to the severity of the disease. In addition, the 2-year time horizon used in the study is not compatible with chronic disease modelling principles despite the fact that guidelines for cost-effectiveness analyses suggest considering a longer time horizon in pharmacoeconomic models for chronic diseases. The last but not the least is that the model used in the study of Azar *et al. *is a simple decision tree model which is not an appropriate model for a chronic multi-stage disease. 

In 2 other studies, teriparatide was compared to no treatment in severe osteoporotic patients and both studies had similar results to those of the current paper (18, 25). In the first study, Ström *et al*. evaluated the cost-effectiveness of teriparatide, PTH (1-84) and no treatment in nine European countries in severe postmenopausal osteoporotic patients. The authors concluded that teriparatide could be a cost-effective strategy depending on the country setting. The cost per QALY gained of postmenopausal women who were under treatment and have had prior vertebral fractures ranged in the base case from “cost saving” in the Scandinavian countries to €15,000 in Italy (25). In the second study, Lundkvist *et al*. offered the same results and emphasized that teriparatide is a cost-effective medicine if it targets appropriate patients (18). 

Biosimilar teriparatide is produced locally in Iran, at a price almost equivalent to 45% of the price of the branded product and this may be one of the main reasons for teriparatide being cost-effective in the country. However, only a small subset of the population with severe osteoporosis with a high risk of fracture are potential candidates for teriparatide. In addition, it is worth mentioning that compared to bisphosphonates, teriparatide had a much higher acquisition cost and it is not a substitute for bisphosphonates. 

This study is subject to several limitations. The most notable of which, and in common with many cost-effectiveness studies, is the generalizability of results to other health care settings. It should be noted that the costs used in the current study are solely relevant to the setting of the Iranian health care system, having been derived from national tariffs, national health care system reference costs, and Iranian studies. 

Secondly, there is not any local clinical trial data available assessing the clinical effectiveness of teriparatide and, consequently, clinical data were derived from the European clinical data which might be different from those in Iran. To address this concern, the authors made substantial efforts to analyse these differences through sensitivity analysis. The same issue is considered about the fracture incidence rate. However, the changes in the clinical effectiveness of teriparatide and baseline fracture incidence were investigated in sensitivity analysis by ± 25% and it was found that the result was not sensitive to this parameter. Finally, we did not model treatment strategies where switching between treatments would be expected.

Using a micro-simulation model can be addressed as a strength for this study. Unlike the Markov cohort model, the micro-simulation can accommodate the possibility to track multiple fractures in an individual patient. In Markov models, patients are distributed in an average number of fractures which implies for all of them. As an additional strength of the study, it should be mentioned that unlike most convenient pharmacoeconomic publications, the adherence of patients to the treatment has been taken into account in this study and the risk of discontinuation of treatment by patients was considered. Incomplete adherence is a major problem in the treatment of osteoporosis, meaning that some patients at a high risk of fracture indicated are not treated. Improving compliance with osteoporosis treatment would make a significant contribution to a further reduction of osteoporosis-related fractures.

Two crucial outcomes were extracted from this study; 1) teriparatide is a cost-effective strategy compared to no treatment in severe PMO populations (total hip T-score -2.5 with previous fracture or T-score -3.0 without prior fracture). 2) However, using teriparatide in other patient populations may not be cost effective (16, 17, 30, 42–44) and a separate investigation is required in this regard.

## Conclusion

This study indicates that compared to no treatment, teriparatide was indicated to be more costly and associated with fewer fractures, more life-years, and more QALYs. The result showed that teriparatide could be considered as a cost-effective medicine in the treatment of severe postmenopausal women, who suffer from a low BMD or have at least one previous fracture over a lifetime horizon, accepting a willingness-to-pay threshold of 2 GDP/capita from the health system perspective. However, more investigation is recommended to assess the cost-effectiveness of teriparatide in comparison with active comparators such as Denosumab in the treatment of patients with severe PMO who have failed or been intolerant of previous osteoporosis treatments.
